# Older Adults' Access to and Satisfaction With Primary Hospitals Based on Spatial and Non-spatial Analyses

**DOI:** 10.3389/fpubh.2022.845648

**Published:** 2022-04-28

**Authors:** Jingyu Yu, Mei-yung Leung, Guixia Ma, Jingcheng Xia

**Affiliations:** ^1^School of Civil Engineering, Hefei University of Technology, Hefei, China; ^2^Department of Architectural and Civil Engineering, City University of Hong Kong, Kowloon, Hong Kong SAR, China; ^3^School of Foreign Studies, Hefei University of Technology, Hefei, China

**Keywords:** accessibility, China, older adults, primary hospitals, two-step floating catchment area method

## Abstract

In order to improve the health and quality of life of older adults, the Chinese government is dedicated to establishing an equilibrium level of primary healthcare services for all communities. However, little attention has been paid to measuring the accessibility of primary hospitals to older adults, nor to understanding the seniors' satisfaction with and needs for primary healthcare services. Therefore, this study sought to investigate the spatial accessibility of primary hospitals to older adults, and also to examine the impact of walking distances on the seniors' satisfaction with their healthcare services. A two-step floating catchment area method was applied to measure the spatial accessibility of primary hospitals to older adults at the level of subdistricts. In order to investigate the actual opinions of older adults and verify the results of spatial analysis, a large-scale questionnaire survey was also conducted. The analyses found that (1) primary hospitals were not equally distributed; (2) most older adults did not have access to primary hospitals within a threshold walking distance of 1,000 m, but they usually could reach a hospital in their subdistrict within a threshold distance of 2,000 m; (3) older adults' satisfaction levels with primary hospitals were significantly different among subdistricts; (4) long walking distances negatively influenced older adults' satisfaction with primary hospitals; (5) the satisfaction of older adults was highest with a threshold distance of 500 m; and (6) a piecewise regression model indicated that older adults' satisfaction with primary hospitals would decrease with an increase in walking distance to the hospital. When the walking distances exceeded 1,000 m, the slope of the linear regression model increased significantly compared with the slope for walking distances less than 1,000 m. By adopting multiple research methods and capturing older adults' behaviors and satisfaction, our results provide (1) data on the importance of accessibility of primary hospitals to older adults, and (2) insights for future planning to achieve equity in primary healthcare and enhance the spatial distribution of primary hospitals.

## Introduction

As a fundamental human right, health plays an essential role in national economic development. In order to improve the health of the entire population, the Chinese government has launched a national agenda, the Healthy China 2030, which aims to increase the average life expectancy to 79 years by 2030 (1). Longevity as a demographic trend has implications in population aging. In fact, China is facing a tremendously large and burgeoning elderly population, with the number of people aged over 65 exceeding 190.6 million in 2020 (2) and increasing to 487 million by 2050, when it will account for 34.9% of the total population (3). Meanwhile, the healthcare needs and demands of older adults are ongoing and continually increase as they age (4).

In China, the government has established a three-tier hospital system, according to the hospitals' service level, facilities, technical skills, and administrative management levels (5). The first-tier hospitals are called primary hospitals and directly offer primary healthcare services, preventive services, and rehabilitation services to community residents. The second-tier hospitals, known as secondary hospitals, provide a higher level of healthcare services to multiple communities. The third-tier hospitals are known as tertiary hospitals, and they provide not only the highest level of healthcare and specialized health services but also medical education and research. The hierarchical medical system is designed to encourage patients to choose hospitals according to their healthcare needs. Unfortunately, Chinese patients often prefer to access higher level hospitals directly, even for simple care needs, and that trend has often resulted in overcrowding and over-utilization of tertiary hospitals (6, 7).

In fact, older adults often suffer from chronic diseases that require access to regular diagnostics and treatment. Primary hospitals are established to provide preventive care and regular diagnosis, monitoring, and treatment for community residents, making them more cost-effective and accessible for older adults than tertiary hospitals are (8). With the added burden of the COVID-19 pandemic, older adults are encouraged to seek basic healthcare services from primary hospitals in order to avoid crowding in tertiary hospitals.

Rational spatial accessibility of healthcare facilities is a major objective of the medical system in many countries. As is mentioned in the Healthy China 2030 plan, China is expected to establish equilibrium primary healthcare services for all communities (9). Primary hospitals are therefore planned on the basis of population scale and service radius. However, previous studies have often focused on the accessibility of tertiary hospitals rather than that of primary hospitals (10), and measuring the accessibility of primary hospitals is still a debated issue. Primary hospitals are developed to serve community residents at subdistrict levels (11), but again, few studies have been conducted to investigate the accessibility of primary hospitals in the subdistricts (12). Moreover, although the spatial accessibility of healthcare has been emphasized by previous studies, older adults' behaviors—-such as their travel modes, needs, and satisfaction, have often been ignored (13). To address that gap, the current paper used both spatial analysis and questionnaire surveys to investigate the accessibility of primary hospitals to older adults and measure those adults' satisfaction with their healthcare services. First, the accessibility of primary hospitals at the subdistrict level was calculated. Next, older adults' satisfaction with their access to primary hospitals was investigated using data collected by questionnaire surveys. Finally, two analyses were compared and policy implications were identified.

This research contributes to the existing literature in two significant ways. First, by the adoption of multiple research methods, our results provide empirical evidence of the accessibility of primary hospitals to older adults by capturing those older adults' behaviors and satisfaction. Second, our results can provide insights for future planning to enhance the spatial distribution of primary hospitals and improve the satisfaction and quality of life of older adults.

## Accessibility

Accessibility was first defined as spatial interactions related to physical areas and locations (14). Accessibility refers to opportunities for access to destinations, and it is associated with the geographic distribution and locations of facilities, street slops, transportation, networks, and so on (15, 16). It can also be measured as the difficulty of gaining access to public resources, in terms of financial support, prices/fees/charges, travel time, safety, services quality, and the like (17). Accessibility is conceptualized into four key elements: land use (i.e., spatial distribution, quantity and quality of facilities), transportation (i.e., transportation systems), time (i.e., available time to utilize facilities), and characteristics of the individual (i.e., income, educational level, age, etc.) (18, 19).

Various measurement methods for evaluating accessibility can be classified as the proximity, cumulative, gravity, utility-based, and space-time prism models. Proximity models are employed to measure accessibility with respect to travel distance, time, or cost. Cumulative models evaluate accessibility as the cumulative number of opportunities available within a threshold distance of time (20). Gravity models provide spatial information from both the demand and supply sides by determining the interaction between two destinations (21). Utility-based models adopt utility theory to model accessibility, which considers the behavioral characteristics of decision-makers (22, 23). Space-time prism models are adopted to measure accessibility from the dimensions of space and time by assessing people's activities in spatial and temporal dimensions (18).

Abundant previous studies have focused on the spatial accessibility of healthcare facilities and hospitals. Huotari et al. (24) adopted a gravity model to investigate the accessibility of tertiary hospitals by using grid-based population data and travel-time estimates. A study conducted by Yiannakoulias et al. (25) employed a gravity-based model for evaluating the spatial accessibility of primary hospitals, accounting for the number of physicians, the population, and the travel costs between an individual's location and the nearest primary hospital. A kernel density two-step floating catchment area (2SFCA) model has been used to calculate the quantity of medical resources in different subdistricts (26). Different travel modes and time thresholds have been applied to evaluate the spatial accessibility of secondary hospitals and tertiary hospitals, and the findings indicated significant spatial differences between different subdistricts. Boisjoly et al. (27) conducted an empirical study to quantify the spatial accessibility of healthcare services for vulnerable populations in metropolitan regions, and they found that vulnerable populations within metropolitan areas had greater access to hospitals via public transport, whereas the accessibility in suburban areas was low. Cheng et al. (28) examined the spatial availability of primary, secondary, and tertiary hospitals for older adults by measuring accessibility with a 2SFCA model, and their results indicated that tertiary hospitals were more unevenly accessible to older adults than primary hospitals were. A study has also been conducted to investigate spatial inequity in hospital accessibility by using a 2SFCA model (12), and it found that low-income neighborhoods experienced relatively lower levels of accessibility of multi-tier hospitals, including primary, secondary, and tertiary hospitals. In consideration of healthcare system reform in China, Xiao et al. (9) proposed three patient-referral models to evaluate heath care accessibility in different scenarios and concluded that patient referrals had prominent effects on the balance of healthcare facilities. A study by Agbenyo et al. (29) adopted both spatial analysis and semi-structured interviews to investigate the accessibility of healthcare facilities and reveal the behaviors and needs of patients. In a consideration of temporal variations in population distribution, Xia et al. (30) proposed a spatial-temporal model for evaluating accessibility of emergency medical services through big GPS data. Wang et al. (31) also adopted big data analysis to measure accessibility of hospitals, with consideration of individuals' preferences for certain hospitals. Individuals' preferences for hospitals were evaluated by taxi trip records in the investigation of hospital access behaviors.

Although previous studies have emphasized hospital accessibility, only a limited number of studies have focused on the accessibility of primary hospitals (10, 25). The research on primary hospital accessibility for older adults is even more insufficient. In fact, older adults are affected by a progressive decline in mobility and health condition, making them increasingly vulnerable to accessibility in their physical environment (32) and more dependent on primary healthcare services, including regular diagnostic services, prescriptions, and examinations (33). Therefore, it is urgent to investigate the accessibility of primary hospitals for older adults. Moreover, most previous studies emphasized the spatial distribution of healthcare services but ignored the actual and subjective opinions of end users. In fact, accessibility to primary hospitals can increase patients' satisfaction in primary hospitals (34). Access to primary hospital influences satisfaction of older adults, but it is often overlooked (32).

Therefore, this study used both spatial and non-spatial analysis to conduct integrated, cross-sectional research on the accessibility of primary hospitals for older adults.

## Methodology

### Study Area

As the capital of Anhui, Hefei is a mega-city with a population of 9.4 million in 2019. In 2020, the population of 65-year-olds and above was more than 1.02 million, which accounted for 12.69% of the entire population. Hefei is a sub-central city of the Yangtze River Delta, and is a national science and education center, manufacturing base, and integrated transportation hub of eastern China. In recent years, Hefei has been one of the most rapidly growing cities in China, with an annual growth rate greater than 4.3%. The total GDP in 2020 exceeded RMB 1,004.5 billion. The administrative division of Hefei consists of four districts (Baohe, Luyang, Shushan, and Yaohai), four counties, and one county-level city.

**Figure 1:**
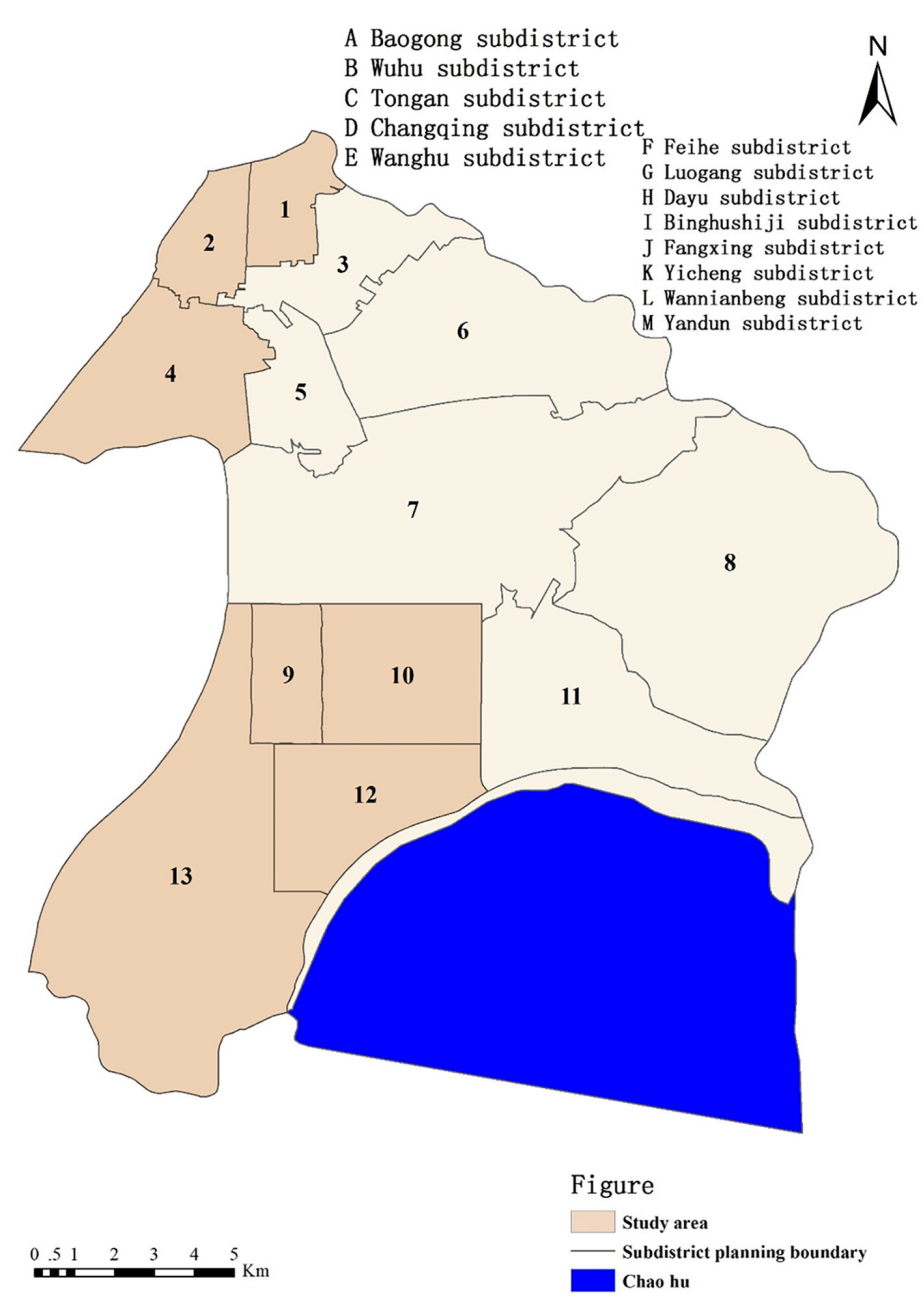
Administration map and study areas of the Baohe District.

In this research, we used Baohe district as a case study (presented in Figure 1). Baohe district has two national model aging-friendly communities. The aging population is biggest in Hefei. The administrative center of the local government is located in Baohe district. The entire area of Baohe district comprises 340 square kilometers and includes Chaohu Lake. The population of Baohe district is 1.42 million, and its GDP exceeds RMB 143.2 billion. There are 13 subdistricts in Baohe district. The research team administered questionnaires in seven of the subdistricts (Baogong, Wuhulu, Changqing, Binhu, Fangxing, Wannianbu, and Yandun subdistricts), where most of the aging population is distributed. Among these subdistricts, Binhu, Fangxing, Wannianbu, and Yandun subdistricts have been classified as new urban regions since 2006. The other three subdistricts (Baogong, Wuhulu, and Changqing subdistricts) are old town areas.

### Framework of Research Methods

In order to investigate older adults' access to primary hospitals, both spatial analyses and non-spatial analyses were conducted. Spatial accessibility of healthcare services and hospitals is usually assessed by using a proximity measure, a cumulative opportunities measure, gravity models, and the two-step floating catchment area (2SFCA) method (12, 21), among which the 2SFCA method has received the most attention. The 2SFCA method was developed by Luo and Wang (35) to assess accessibility from both the demand and supply sides of the question. The method calculates accessibility in two steps. First, the supply-to-demand ratio is estimated for each location by distributing the number of resources to population points within the catchment area. The supply-to-demand ratio is then assigned to the entire catchment area. In the second step, the supply-to-demand ratios of all providers estimated in the first step are summed and each population point is assigned an accessibility value.

The original 2SFCA method is used to hypothesize the same supply-to-demand ratios for population points, ignoring the impact of distances on individual mobility preferences. In order to model the effects of distances, an enhanced 2SFCA method (i.e., Ga2SFCA) was proposed by Luo and Qi (36) that adopted a Gaussian function to measure different distance decay values. We adopted that enhanced Ga2SFCA method to calculate the spatial accessibilities of primary hospitals for older adults in Hefei at the subdistrict level.

The calculations in the Ga2SFCA method comprise two steps. The first step is to calculate the service coverage of primary hospitals. For each primary hospital at location *j*, all demand points (i.e., residential communities *k*) are searched within the threshold distance *d*_0_. The catchment area is determined by the location *j* and the threshold distance *d*_0_. The supply-to-demand ratio *R*_*j*_ of a primary hospital at location *j* within the catchment area is computed by using the following equation:


(1) $$\begin{array}{l} R_{j}=\frac{S_{j}}{\sum_{k\in \left(d_{kj}\leq d_{0}\right)}f\left(d_{kj}\right)P_{k}} \end{array}$$

where *R*_*j*_ is the supply-to-demand ratio at location *j*. The term *S*_*j*_ is the total supply of primary hospitals at location *j* and is measured by the number of medical staff. In previous studies on the accessibility of hospitals, the supply of hospitals was often evaluated using the number of beds. However, primary hospitals mainly undertake basic medical services and not in-patient services. Hence, for our current study we adopted the number of medical staff to evaluate the supply side of primary hospitals. The term *P*_*k*_ is the number of aging populations at residential community *k* within the threshold distance *d*_*j*_, thus representing the quantity of demand; *d*_*kj*_ is the distance between location *j* and residential community *k*.

The second step is to sum the services that each residential community receives from primary hospitals. Each elderly population point in residential community *i* is set as a searching center, and a threshold distance *d*_0_ is set as the searching radius. The catchment area is determined by each searching center *i* and threshold distance *d*_0_. Accessibility *A*_*i*_ is measured by summing all supply-to-demand ratio *R*_*j*_ values for which the location of a primary hospital falls within the catchment area centered in residential community *i*. The values of *R*_*j*_ were obtained in the first step. Accessibility *A*_*i*_ is calculated as:


(2) $$\begin{array}{l} A_{i}=\underset{j\in \left(d_{ij}\leq d_{0}\right)}{\sum }f\left(d_{ij}\right)R_{j}, \end{array}$$

where *A*_*i*_ is the accessibility of primary hospitals in residential community *i*, *d*_*ij*_ is the distance between residential community *i* and each primary hospital at location *j*, and *f*(*d*) in the two equations is the impedance function defined next in equation (3), which is a Gaussian function. The accessibility calculation assumes that even within the same catchment, older adults prefer to choose closer primary hospitals rather than other, more distant ones (37). Thus, impedance *f*(*d*) is:


(3) $$\begin{array}{l} f\left(d_{ij}\right)=\{\begin{array}{ll} \frac{\exp\left(-\frac{1}{2}{\left(\frac{d_{ij}}{d_{0}}\right)}^{2}\right)-\exp\left(-\frac{1}{2}\right)}{1-\exp\left(-\frac{1}{2}\right)}, & d_{ij}\leq d_{0}\\ 0, & d_{ij}>d_{0} \end{array} \end{array}$$

In addition to spatial analyses, the study used questionnaire surveys to collect older adults' opinions about their satisfaction with the accessibility of primary hospitals. The survey consisted of three major sections: (1) background demographic information about the older adults (e.g., age, education, income, marriage, health conditions and mobility, etc.); (2) frequency of use by and walking distances for respondents to access primary hospitals; and (3) the respondents' degree of satisfaction with the accessibility of primary hospitals. Frequency of use of primary hospitals was measured with four levels: more than once per week, 1–2 times per month, 1–3 times per year, and “seldom.” Walking distances for the older adults to the nearest primary hospitals were also measured with four levels: within 500 m, within 1,000 m, within 1,500 m, and within 2,000 m. A five-point Likert-type scale was used to measure the respondents' level of satisfaction with the accessibility of primary hospitals, with 1 = very dissatisfied; 2 = dissatisfied; 3 = neutral; 4 = satisfied; and 5 = very satisfied.

Several statistical methods were used to analyze the data collected from the questionnaire and investigate actual behaviors of older adults, which could not be directly determined from spatial analysis. An analysis of variance (ANOVA) was applied to investigate the differences between hospital accessibility and the satisfaction of the older adults with their primary hospitals. Multiple regression analysis was adopted to identify the critical factors that influenced the older adults' level of satisfaction with the accessibility of primary hospitals.

### Research Data

**Figure 2:**
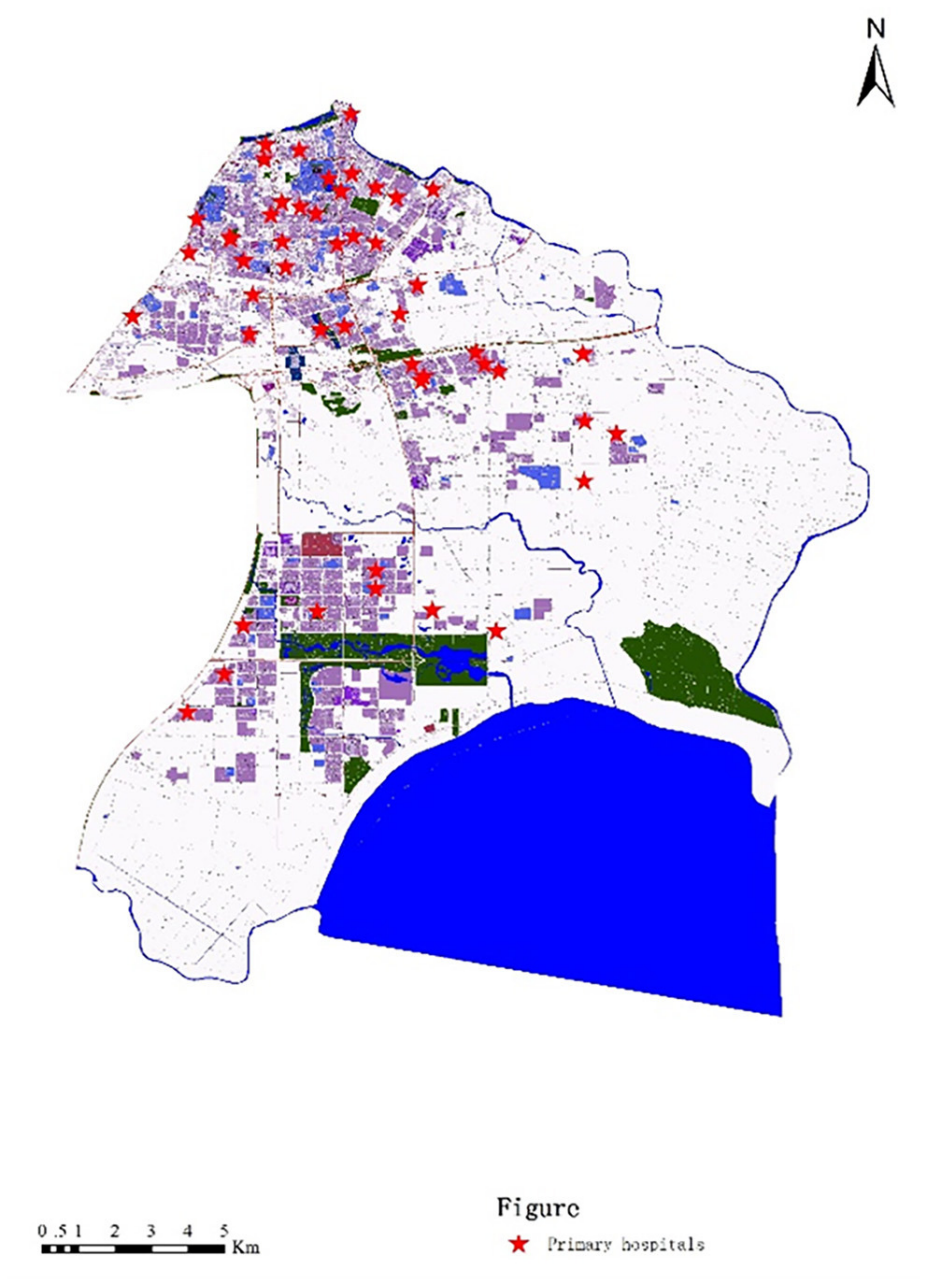
Locations of primary hospitals in the study areas.

**Figure 3:**
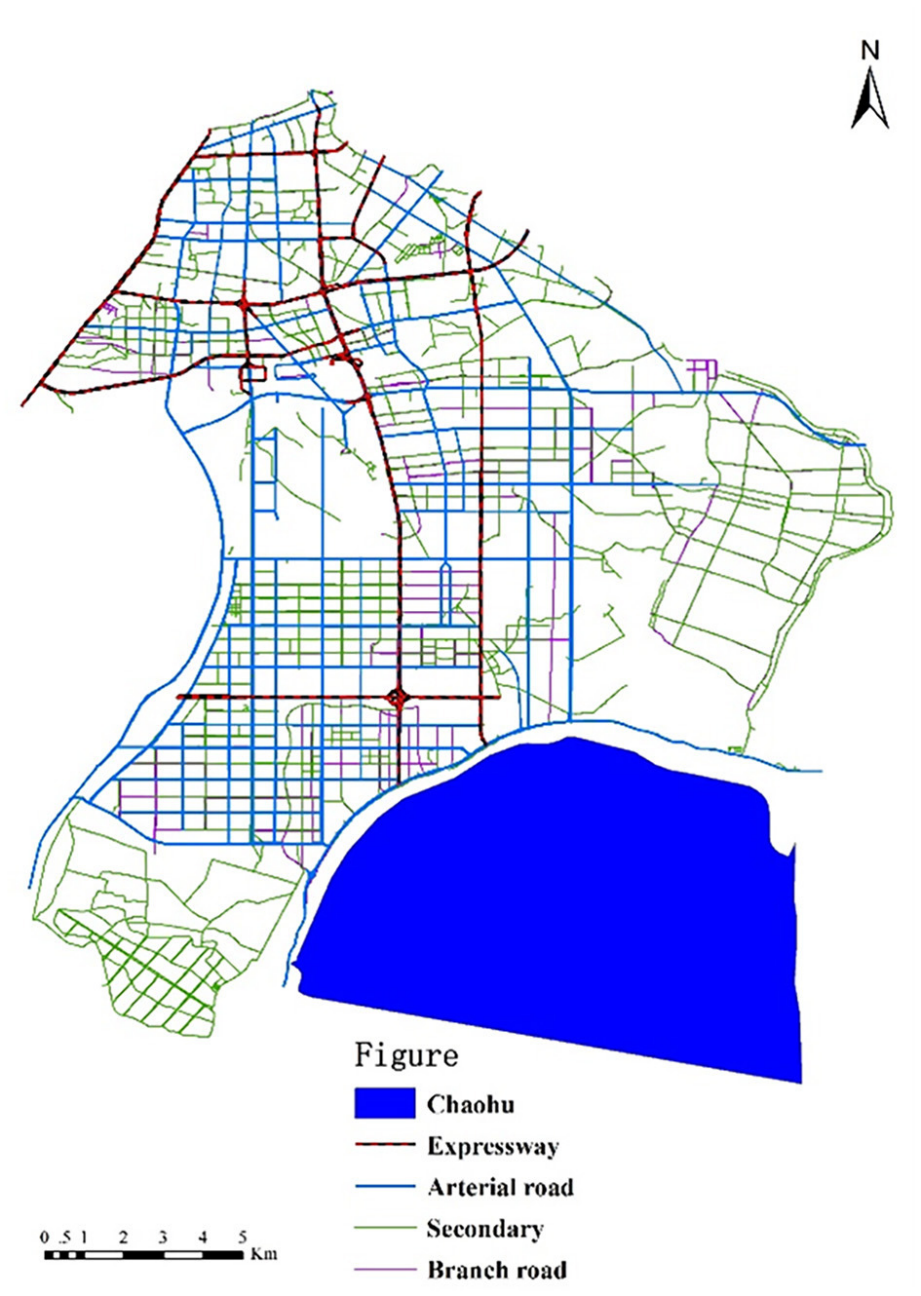
Road networks of the study areas.

In order to measure the spatial accessibility of primary hospitals, both the supply of primary hospitals and the demand for them were evaluated. For the supply aspect, data on the locations of primary hospitals, the numbers of medical staff, and the road networks of the subdistricts were gathered for the study. Locations of primary hospitals were gathered from the official website of Hefei Civil Affairs. The study areas comprised seven subdistricts in Hefei, and those subdistricts had 48 primary hospitals (Figure 2). The numbers of medical staff in the primary hospitals were obtained from local health bureaus. The road networks were extracted from Baidu Maps, which is the largest electronic map service in China (Figure 3).

The demand for primary hospitals focuses on the needs of older adults. Hence, data on the elderly population and their residential communities' locations were analyzed. Population data are only available at the district level in Hefei, so to analyze the elderly population at the subdistrict level, it was hypothesized that the older adults were symmetrically distributed in the study's seven subdistricts. We used luminous remote-sensing data to measure population data for the subdistricts, with the following steps.

Step 1: Collect luminous remote-sensing images from Luojia No. 1.

**Figure 4:**
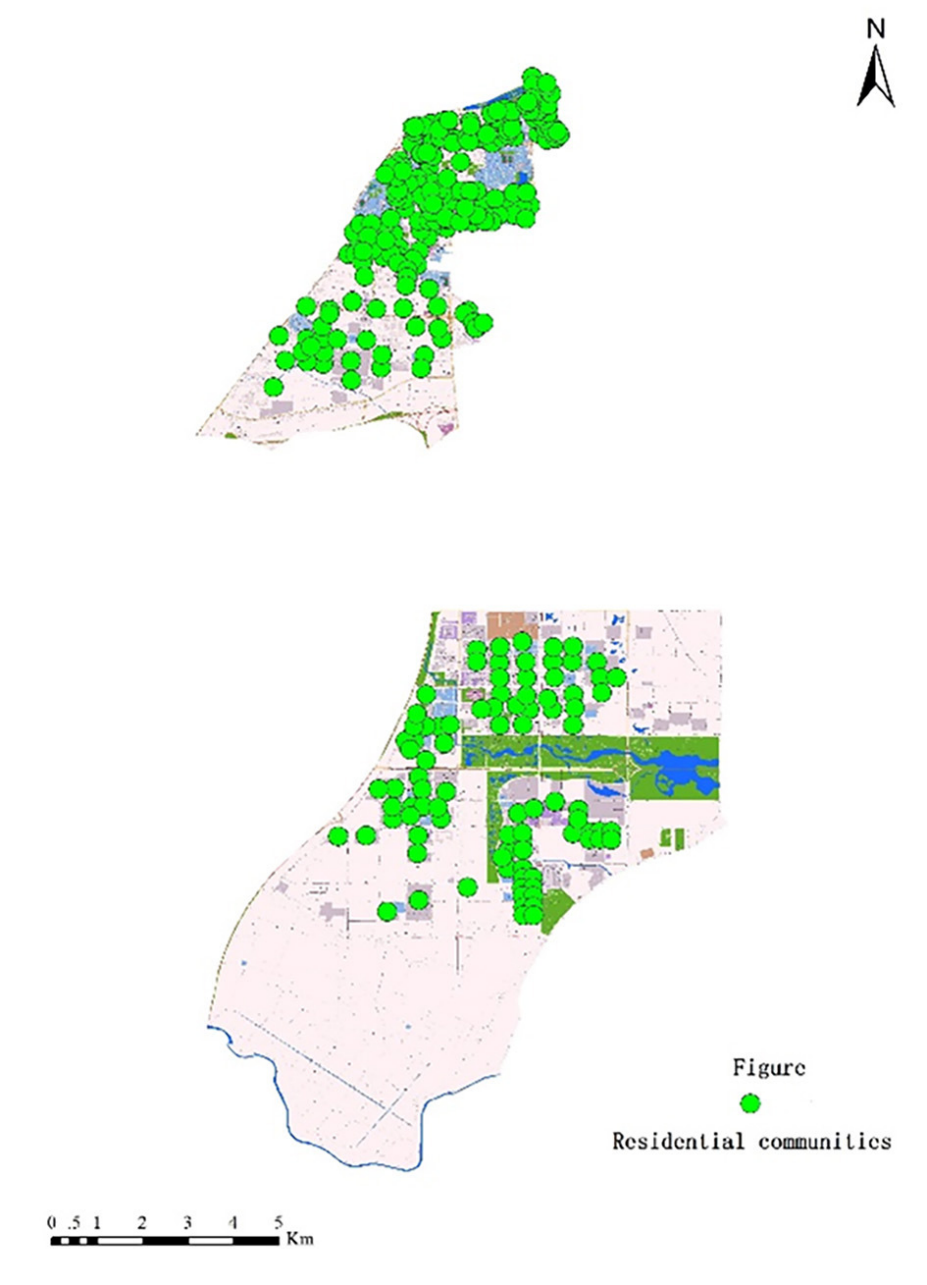
Locations of residential communities in the study areas.

Step 2: Extract locations of residential communities from Baidu Maps (Figure 4).

Step 3: Analyze luminous remote-sensing images in ArchGIS by obtaining the gray values *D*_*x*_ of residential communities in the seven subdistricts.

Step 4: Sum the gray values to determine the total gray value *D*_*Ni*_ of subdistrict *i*.

Step 5: Establish a model for calculating population data *P*_*i*_ of subdistrict *i*. The fitting model in the current study was $P_{i}=0.5684\times D_{Ni}^{0.7116}$ (38).

Step 6: Estimate the elderly population by using population data *P*_*i*_ multiplied by the rate of aging in the population in Hefei (i.e., 12.69%).

**Figure 5:**
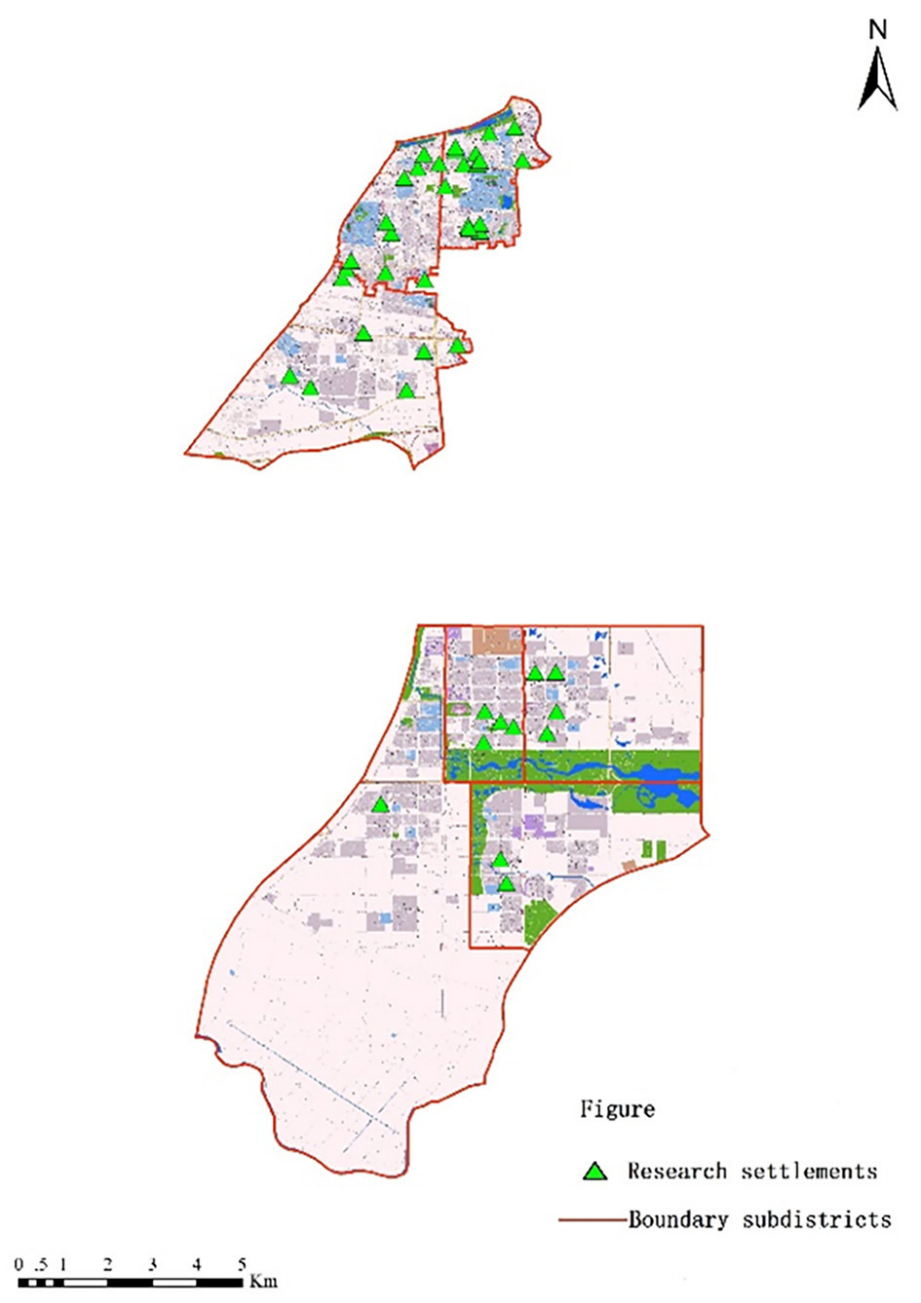
Distribution of questionnaire surveys in the study areas.

In order to conduct our non-spatial analysis, questionnaires were administered to collect the subjective opinions of older adults in the seven study subdistricts (Figure 5). Respondents were distributed in different residential communities that were near primary hospitals. Purposive sampling was applied to select appropriate respondents who: (1) were over 60 years of age at the time they took the survey; (2) had accessed primary hospitals at least once within a year; and (3) had sufficient cognitive and linguistic abilities to understand and respond to the questionnaire. There were 1,426 older respondents in total, 633 of which were males (44.4%) and 793 were females (55.6%). A total of 50.6% of the respondents were age 60–69, 37.1% were 70–79, 11.4% were 80–90, and 0.9% were over 90. Most of the older respondents were not well educated (beyond junior high school), and only 4.1% had attended college. Nearly half of the elderly respondents (47%) were self-reported as healthy, but 34.2% of respondents were generally unhealthy and 18.8% of respondents were very unhealthy. Only a very few respondents (3.2%) had mobility problems, and the remaining seniors had good mobility. Nearly all of the elderly respondents suffered from chronic diseases, including hypertension (34.6%), diabetes (22.1%), heart disease (10.9%), chronic bronchitis (11.2%), chronic gastritis (10.2%), and arthritis (19.5%). There were 15.9% who reported a monthly income of less than $141.50, 22.5% reported $141.50–353.75, 32.3% had an income of $353.75–707.50, and 29.4% of the respondents received over $707.50.

## Results

### Spatial Accessibility of Primary Hospitals

**Figure 6:**
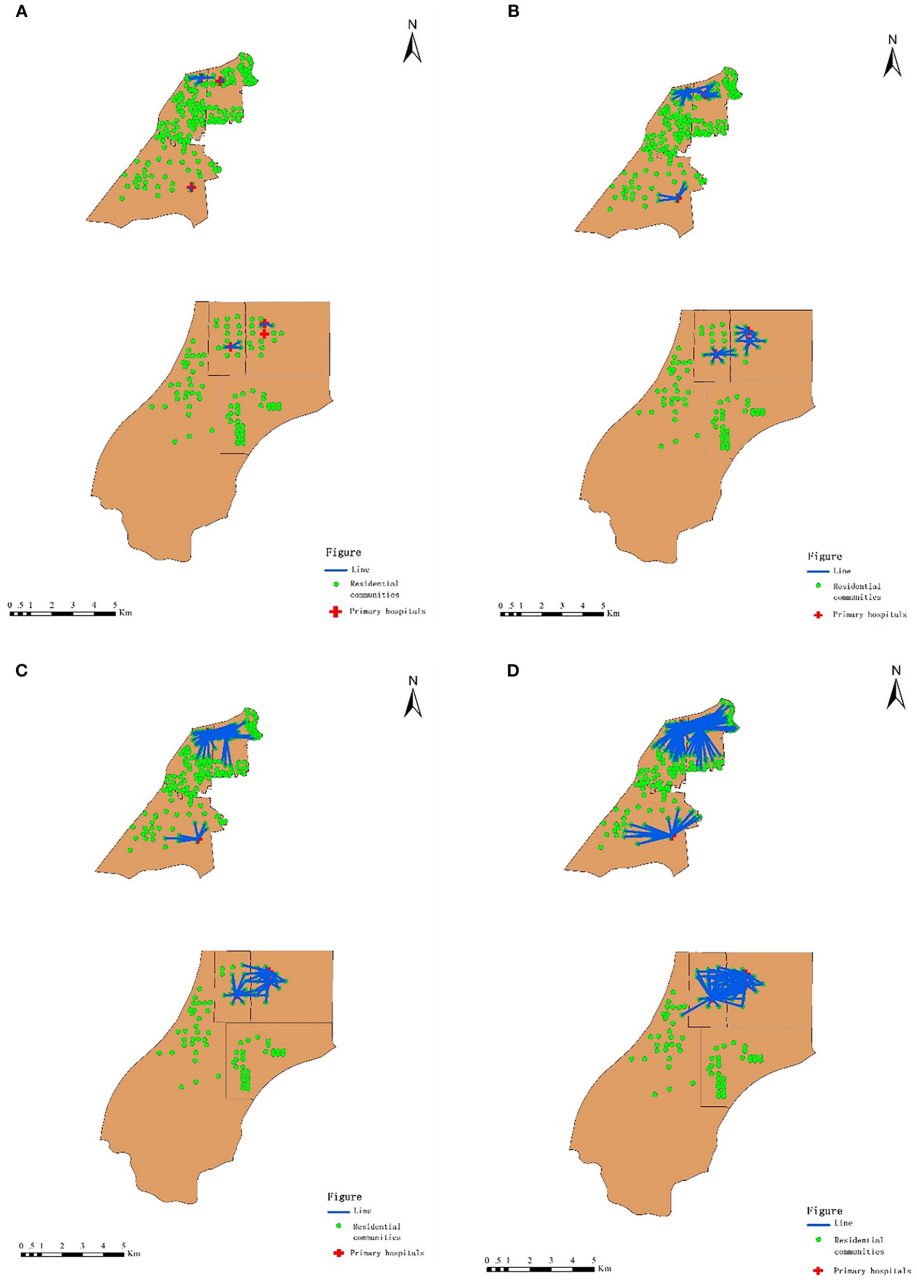
Distances between residential communities of older adults and primary hospitals. **(A)** Threshold Walking Distance ≤500 m. **(B)** Threshold walking distance ≤1,000 m. **(C)** Threshold walking distance ≤1,500 m. **(D)** threshold walking distance ≤2,000 m.

In order to measure the accessibility of primary hospitals for older adults, four threshold distances-−500 m, 1,000 m, 1,500 m, and 2,000 m—were determined (Figure 6). Most of China's older adults rely on walking in their daily lives (39). The mobility scopes of older adults are often restricted into a 2,000 m radius centered by their residential communities. Hence, the threshold distance should not be longer than 2,000 m.

**Figure 7:**
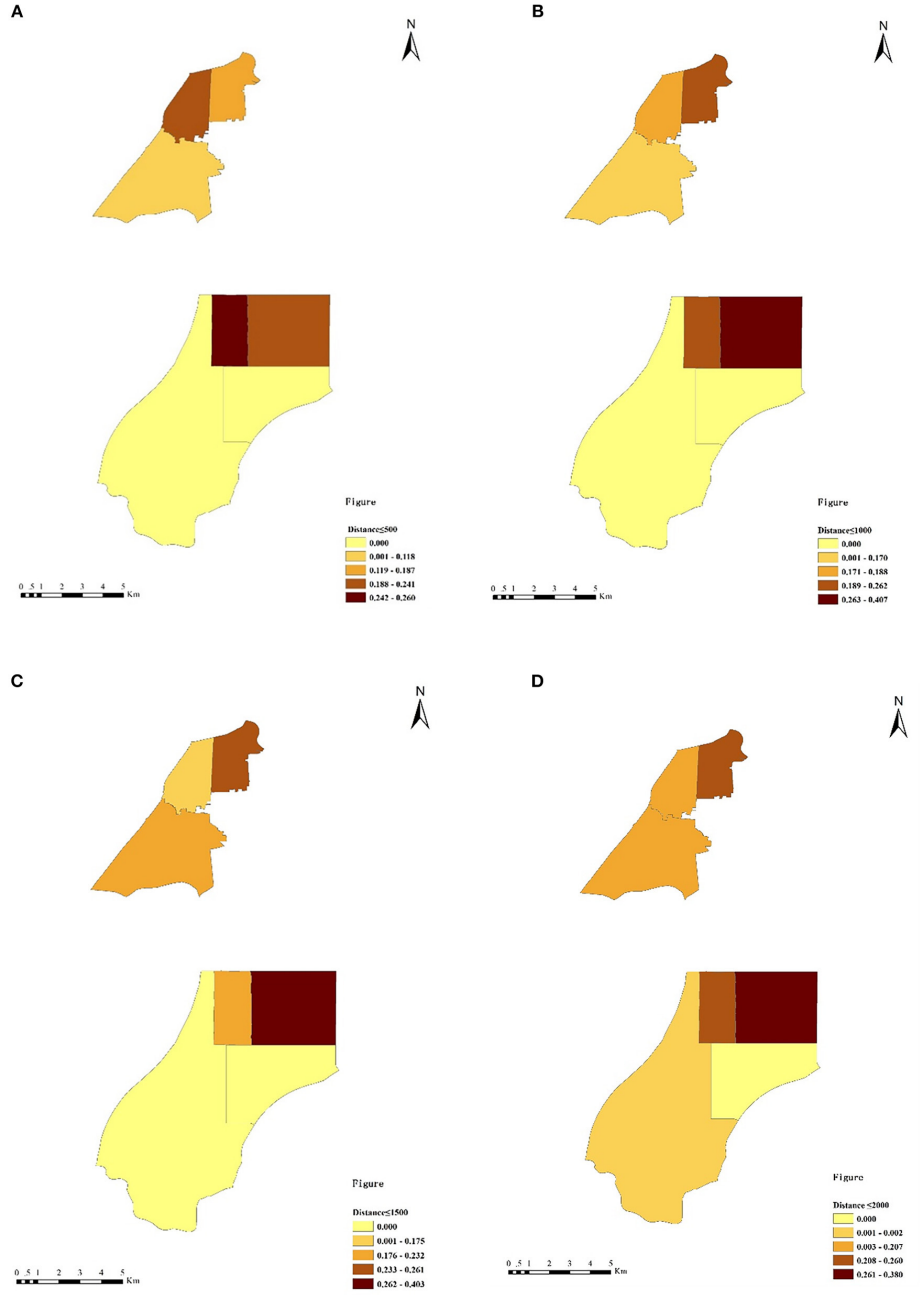
The accessibility values of primary hospitals at different threshold distances. **(A)** Threshold walking distance of 500 m. **(B)** Threshold walking distance of 1,000 m. **(C)** Threshold walking distance of 1,500 m. **(D)** Threshold walking distance of 2,000 m.

The data for the geographic accessibility of primary hospitals for older adults are presented in Table 1. Figure 7 displays how the spatial patterns of accessibility differed among the seven subdistricts. In new urban areas, the level of accessibility of primary hospitals was quite unevenly distributed. Primary hospitals were relatively more accessible surrounding the center of new urban areas than in the other types of areas (e.g., Binhu and Fangxing subdistricts). In the suburbs (e.g., Yandun and Wannianbu), however, the accessibility of primary hospitals was quite low—nearly zero.

**Table 1:** Accessibility values of primary hospitals in different subdistricts.

**Subdistricts**	**Accessibility**			
	**500 m**	**1,000 m**	**1,500 m**	**2,000 m**
**Old town areas**				
Changqing	0.118	0.170	0.207	0.207
Baogong	0.187	0.262	0.261	0.260
Wuhulu	0.233	0.188	0.175	0.181
**New urban areas**				
Binhu	0.260	0.225	0.232	0.256
Fangxing	0.241	0.407	0.403	0.380
Wannianbu	0	0	0	0
Yandun	0	0	0	0.001

In old town areas, the equilibrium distribution of the accessibility of primary hospitals was relatively better. The accessibility values of primary hospitals in three subdistricts (Baogong, Wuhulu, and Changqing) were similar. Within the threshold distance of 500 m, the accessibility of primary hospitals in Wuhulu subdistrict (i.e., 0.233) was higher than that in Baogong and Changqing subdistricts (i.e., 0.187 and 0.118, respectively). The accessibility of primary hospitals in Wuhulu subdistrict decreased along with the increasing threshold distance, whereas the accessibility of primary hospitals in Baogong and Changqing subdistricts increased when the threshold distance was greater than 500 m. The accessibility values of primary hospitals in Baogong subdistrict were highest at the distances of 1,000 m, 1,500 m, and 2,000 m, at 0.262, 0.261, and 0.260, respectively.

### Respondents' Perspectives on the Accessibility of Primary Hospitals

The data gathered from our questionnaire surveys provided a good foundation for understanding older adults' perspectives on the accessibility of primary hospitals. The ANOVA results indicated that older adults in different subdistricts had different satisfaction levels with the accessibility of primary hospitals (i.e., the *F* value was 13.989 at a significance level of 0.01, as is listed in Table 2). Older adults in Binhu subdistrict reported a significantly higher satisfaction level with the accessibility of primary hospitals, compared with the senior respondents in other subdistricts (i.e., the mean value of satisfaction was 4.090 in Binhu subdistrict). The satisfaction levels of older adults in Wannianbu and Yandun subdistricts (with mean values of 3.306 and 3.330, respectively) were significantly lower than those in other subdistricts. Those results were consistent with the spatial accessibility analysis, which indicated an unbalanced distribution of primary hospitals in new urban areas. In the old town areas, seniors in Baogong subdistrict had significantly higher satisfaction with the accessibility of primary hospitals (with a mean value of 3.824) than the older adults in Wuhulu subdistrict did (with a mean value of 3.628).

**Table 2:** ANOVA analysis of subdistricts and older adults' levels of satisfaction with the accessibility of primary hospitals.

**Subdistricts**	**Mean value**	**Mean differences**							***F* value**
		**Changqing**	**Baogong**	**Wuhulu**	**Binhu**	**Fangxing**	**Wannianbu**	**Yandun**	
Changqing	3.686	0	−0.138	0.058	−0.404^**^	0.026	0.380^**^	0.356^**^	13.989^**^
Baogong	3.824	0.138	0	0.196^*^	−0.266^**^	0.164	0.518^**^	0.494^**^	
Wuhulu	3.628	−0.058	−0.196^*^	0	−0.462^**^	−0.032	0.322^**^	0.298^**^	
Binhu	4.090	0.404^**^	0.266^**^	0.462^**^	0	0.430^**^	0.784^**^	0.760^**^	
Fangxing	3.660	−0.026	−0.164	0.032	−0.430^**^	0	0.354^**^	0.330^**^	
Wannianbu	3.306	−0.380^**^	−0.518^**^	−0.322^**^	−0.784^**^	−0.354^**^	0	−0.024	
Yandun	3.330	−0.356^**^	−0.494^**^	−0.298^**^	−0.760^**^	−0.330^**^	0.024	0	

* *means significant at the 0.05 level*;

** *means significant at the 0.01 level*.

In order to investigate the impact of walking distances on older adults' satisfaction with the accessibility of primary hospitals, an ANOVA analysis was conducted by setting satisfaction as the dependent variable and walking distances as an independent variable. The results presented in Table 3 indicated that (1) walking distances significantly influenced older adults' satisfaction with the accessibility of primary hospitals (i.e., the *F* value was 20.427 at a significance level of 0.01); (2) when walking distances to primary hospitals reached 2,000 m, the older adults had a significantly lower satisfaction level (mean value of 3.226) than the other older adults did with shorter distances; (3) the satisfaction level for the accessibility of primary hospitals at a distance of 500 m (mean value of 4.370) was significantly higher than that at a distance of 1,000 m (mean value of 3.763).

**Table 3:** ANOVA analysis of walking distances and older adults' satisfaction with the accessibility of primary hospitals.

**Walking distances**	**Mean value of satisfaction**	**Mean differences**				***F* value**
		**500 m**	**1,000 m**	**1,500 m**	**2,000 m**	
500 m	4.370	0	0.607^**^	0.495	1.144^**^	20.427^**^
1,000 m	3.763	-0.607^**^	0	−0.112	0.537^**^	
1,500 m	3.875	−0.495	0.112	0	0.649^**^	
2,000 m	3.226	-1.144^**^	-0.537^**^	-0.649^**^	0	

* means significant at the 0.05 level;

** *means significant at the 0.01 level*.

A multiple linear regression model was applied to further investigate the impact of subdistricts and walking distances on older adults' satisfaction with the accessibility of primary hospitals. As illustrated in Table 4, the results indicated that only walking distances exerted a significant, negative impact on older adults' satisfaction with the accessibility of primary hospitals, with a variance of 10.8%. The impact of subdistricts on older adults' satisfaction with the accessibility of primary hospitals was not significant, according to the results of our multiple linear regression model.

**Table 4:** Multiple linear regression results.

**Dependent variable**	**Independent variables**	***B***	***t***	**Sig.*t***	***R* ^**2**^**	***F***	**Sig.*F***
Satisfaction	Constant	4.603	25.471	0.000	0.108	28.653	0.000
	Subdistricts	−0.017	−0.730	0.466			
	Walking distances	−0.329	−7.558	0.000			

According to the results of our ANOVA analysis, the mean values of older adults' satisfaction with the accessibility of primary hospitals were significantly different at different distances. Hence, a piecewise linear regression model was adopted to predict the complicated impact of walking distances on older adults' satisfaction with primary hospital accessibility. The regression model was set with the following equation.


$$Satisfaction=\alpha +\beta_{1}\times d\times I_{d\leq d_{0}}+\beta_{2}\times d\times {I^{\prime }}_{d>d_{0}},$$

where β_1_ is the coefficient of walking distances when they are smaller than *d*_0_;

β_2_ is the coefficient of walking distances when they are larger than *d*_0_;

α is a constant of the regression model;

*d* is the walking distance for older adults to a primary hospital;

*d*_0_ refers to threshold distances at 500 m, 1,000 m, 1,500 m;

when *d* ≤ *d*_0_, *I* =1, when *d* > *d*_0_, *I* =0; and

when *d* ≤ *d*_0_, *I*′ =0, when *d* > *d*_0_, *I*′ =1.

The results of piecewise linear regression models are given in Table 5. Three piecewise linear regression models were predicted, and they adopted three threshold distances: 500 m, 1,000 m, and 1,500 m. The *R*^2^ for model III (i.e., 0.098) was lower than that of the multiple linear regression model that was listed in Table 4. The impact of walking distances on older adults' satisfaction was also preposterous in model III, in which the coefficient of walking distance was positive (i.e., β_1_ = 3.916). Hence, model III was proved to be invalid. The *R*^2^ values for model I and model II (0.114 and 0.198, respectively) were both higher than that in the original regression model, which indicated a better fit. Moreover, the coefficients of walking distances were negative, which were consistent with the previous regression model in Table 4. We believe that model I and model II were valid, and model II had a better fit, with a variance of 19.8%. Thus, the final regression model was predicted to be:


$$\begin{array}{l} Satisfaction=3.922-0.649\times d\times I\left(d\leq 1000\right)\\ \;-0.823\times d\times I^{\prime }\left(d\;>1000\right). \end{array}$$

The above piecewise regression model indicated that older adults' satisfaction with the accessibility of primary hospitals would decrease in conjunction with the increase in walking distances. When the walking distances exceeded 1,000 m, the slope of the linear regression model increased, thus indicating that older adults' satisfaction with primary hospitals' accessibility rapidly decreased when the walking distances to primary hospitals were longer than 1,000 m.

**Table 5:** Piecewise linear regression models.

	**Dependent variables**	**Independent variables**	**β**	**Lower confidence limit**	**Upper confidence limit**	***R* ^**2**^**
I	*d*_0_ = 500 m Satisfaction	β_1_	−0.284	−0.394	−0.173	0.114
		β_2_	−1.368	−0.598	−2.479	
		α	4.370	4.028	4.712	
II	*d*_0_ = 1000 m Satisfaction	β_1_	−0.649	−1.107	−0.192	0.198
		β_2_	−0.823	−0.012	−1.635	
		α	3.922	3.746	4.099	
III	*d*_0_ = 1500 m Satisfaction	β_1_	3.916	3.752	4.080	0.098
		β_2_	−294.263	−1.031	587.495	
		α	74.372	−44.555	193.299	

## Discussion

The results of accessibility analysis indicated that the spatial distribution of primary hospitals in new urban areas was unbalanced—-a finding that was consistent with those of previous studies [e.g., (40)]. Older adults in the central subdistricts of new urban areas (i.e., Binhu and Fangxing subdistricts) had better access to primary hospitals than did seniors from suburban neighborhoods such as in Wannianbu and Yandun subdistricts. That improved access might be attributed to the fact that the supply of primary hospitals has lagged behind the development of ongoing urban sprawl. Although rapid urbanization expanded the vast suburban areas of Wannianbu and Yandun subdistricts, primary hospitals in these two subdistricts were seriously insufficient, and that resulted in the low accessibility of nearly zero. The accessibilities of the primary hospitals in old town areas were similar to each other, indicating a relatively equal spatial distribution (28). The explanation for such distributions may be that subdistricts in old town areas are usually high-density communities, and primary hospitals are provided nearby residential communities. Hence, it is easier for older adults to gain access to primary hospitals in old town areas than it is in new urban and suburban areas.

Non-spatial analysis can be used to verify the results of our accessibility analysis. The ANOVA results also showed that older adults in different subdistricts reported different levels of satisfaction with the primary hospitals, which was consistent with other studies (27). Older adults living in Binhu subdistrict had a significantly higher satisfaction level than did those from other subdistricts, which conformed to the relatively high accessibility value of Binhu subdistrict. It is interesting to note that satisfaction with the primary hospitals in Fangxing subdistrict was only higher than that for the other two subdistricts in new urban areas. Although the accessibility of primary hospitals in Fangxing subdistrict was highest at the threshold distances of 1,000 m, 1,500 m and 2,000 m, the satisfaction level with the primary hospital was not prominent. It might reflect that the allocation of primary hospitals in Fangxing subdistrict failed to satisfy older adults in the areas within 500 m, and that allocation then led to intra-district inequity.

Walking distances significantly influenced the satisfaction of older adults with their primary hospitals, a finding that was also proved by previous studies (32). The satisfaction with primary hospitals decreased when the walking distances to them increased. Older adults reported a significantly highest satisfaction with primary hospitals at the threshold distance of 500 m. When the walking distances to primary hospitals exceeded 1,000 m, the older adults' level of satisfaction rapidly declined, as is indicated by the piecewise regression model. The results of network analysis indicated that most of the primary hospitals could not be accessed by older adults within a distance of 1,000 m, although that might be attributed to the cut-off point of piecewise regression model having been set at the distance of 1,000 m. Older adults often suffer from mobility problems, and the long walking distances (e.g., over 1,000 m) might restrict them to using primary healthcare services, which decreases their satisfaction with primary hospitals (41). Furthermore, they might need to rely on public transportation when the distances to primary hospitals are longer than 1 kilometer.

The study's findings are important for policymakers in their efforts to optimize the spatial distribution and supply of primary hospitals and thereby improve their accessibility to and satisfaction in older adults. In order to reduce spatial disequilibrium, tailor-made strategies are suggested for specific subdistricts, depending on actual situations. It is recommended that additional primary hospitals be provided in the suburban areas where primary hospitals are inadequate. If a district has a high level of overall accessibility but a relatively low level of user satisfaction, intra-district inequity should be considered. Primary hospitals should be provided on the basis of the distribution of older adults and their residential communities. Primary hospitals should be supplied in accord with walking distances and the mobility of older adults. It is better to provide primary hospitals within walking distances of 1,000 m or less around older adults' residential communities.

Although this pilot study generated interesting findings, certain limitations should be noted. First, because data for aging populations at the subdistrict level were lacking, we adopted luminous remote sensing data and hypothesized that older adults are symmetrically distributed. That limitation could lead to a deviation from the actual situations of accessibility for older adults. Hence, we also conducted large-scale questionnaires to explore the opinions and satisfaction of older adults regarding primary hospitals. Second, it is suggested that the supply capacity of primary hospitals be further investigated by evaluating multiple factors, including the number of beds, medical facilities, and medical staff. Third, socioeconomic attributes are recommended for further study in order to investigate spatial equity and spatial disparity among different groups of elders. As the predicting accuracy of current results is relatively low, it is strongly recommended to adopt polynomial function and other regression methods to analyze the impact of different factors on accessibility to and satisfaction on primary hospitals. Additionally, it is suggested that bid data methods be adopted and the GPS dataset of older adults be employed to analyze real time accessibility with a spatial-temporal model.

## Conclusions

With today's rapid demographic shift toward an aging society, the demands of older adults on primary healthcare services are constantly increasing. Older adults often suffer from chronic diseases and thus require access to regular diagnostics and treatment. Primary hospitals, which provide mainly preventive care and regular diagnostics, monitoring, and treatment for community residents, are more cost-effective and accessible for older adults than tertiary hospitals are. However, previous studies often have focused on the accessibility of tertiary hospitals instead of that of primary hospitals. Little attention has been paid to measuring the accessibility of primary hospital care for older adults nor to understanding their satisfaction with and need for primary healthcare services. Therefore, both spatial analysis and questionnaire surveys were conducted to investigate the accessibility of primary hospitals to older adults and to examine the impact of walking distances on their satisfaction with their access to healthcare.

Several significant findings resulted. First, primary hospitals were not equally distributed. Accessibility of primary hospitals in subdistricts near the centers of new urban areas was better than that in subdistricts of old town areas and suburban areas. Second, within the threshold walking distance of 1,000 m, most older adults did not have access to primary hospitals, but most primary hospitals could be reached within the threshold distance of 2,000 m, except for those in suburban areas. Third, it was indicated that older adults' satisfaction with primary hospitals would decrease in conjunction with an increase in walking distances. When the walking distances were over 1,000 m, the slope of the linear regression model increased significantly. The findings will be useful for policymakers in their efforts to optimize the spatial distribution and supply of primary hospitals, thereby improving hospital accessibility for older adults and enhancing their satisfaction. These results are expected to provide insights for future urban planning that seeks to enhance the spatial distribution of primary hospitals and improve older adults' satisfaction with healthcare.

## Data Availability Statement

The raw data supporting the conclusions of this article will be made available by the authors, without undue reservation.

## Ethics Statement

Ethical review and approval was not required for the study on human participants in accordance with the local legislation and institutional requirements. The patients/participants provided their written informed consent to participate in this study.

## Author Contributions

JY: conceptualization, methodology, software, and writing. M-yL: conceptualization, reviewing, and editing. GM: data collection. JX: reviewing and editing. All authors contributed to the article and approved the submitted version.

## Funding

The work described in this paper was fully supported by a grant from the Social Science Funds of Anhui Province (Project No. AHSKY2019D031) and the Research Funds of Hefei University of Technology (JZ2020HGTB0068).

## Conflict of Interest

The authors declare that the research was conducted in the absence of any commercial or financial relationships that could be construed as a potential conflict of interest.

## Publisher's Note

All claims expressed in this article are solely those of the authors and do not necessarily represent those of their affiliated organizations, or those of the publisher, the editors and the reviewers. Any product that may be evaluated in this article, or claim that may be made by its manufacturer, is not guaranteed or endorsed by the publisher.
